# Canine Parvovirus VP2 Protein Expressed in Silkworm Pupae Self-Assembles into Virus-Like Particles with High Immunogenicity

**DOI:** 10.1371/journal.pone.0079575

**Published:** 2014-01-17

**Authors:** Hao Feng, Gui-qiu Hu, Hua-lei Wang, Meng Liang, Hongru Liang, He Guo, Pingsen Zhao, Yu-jiao Yang, Xue-xing Zheng, Zhi-fang Zhang, Yong-kun Zhao, Yu-wei Gao, Song-tao Yang, Xian-zhu Xia

**Affiliations:** 1 Institute of Military Veterinary, Academy of Military Medical Sciences, Changchun, Jilin Province, China; 2 Agricultural Division, College of Animal Science and Veterinary Medicine, Jilin University, Changchun, Jilin Province, China; 3 College of Animal Science and Technology, Jilin Agricultural University, Changchun, Jilin Province, China; 4 Institute of Laboratory Animal Sciences, Chinese Academy of Medical Sciences & Peking Union Medical College, Bejing, China; 5 Biotechnology Research Institute, Chinese Academy of Agricultural Sciences, Beijing, China; St. Jude Children's Research Hospital, United States of America

## Abstract

The VP2 structural protein of parvovirus can produce virus-like particles (VLPs) by a self-assembly process *in vitro*, making VLPs attractive vaccine candidates. In this study, the VP2 protein of canine parvovirus (CPV) was expressed using a baculovirus expression system and assembled into parvovirus-like particles in insect cells and pupae. Electron micrographs of VLPs showed that they were very similar in size and morphology when compared to the wild-type parvovirus. The immunogenicity of the VLPs was investigated in mice and dogs. Mice immunized intramuscularly with purified VLPs, in the absence of an adjuvant, elicited CD4^+^ and CD8^+^ T cell responses and were able to elicit a neutralizing antibody response against CPV, while the oral administration of raw homogenates containing VLPs to the dogs resulted in a systemic immune response and long-lasting immunity. These results demonstrate that the CPV-VLPs stimulate both cellular and humoral immune responses, and so CPV-VLPs may be a promising candidate vaccine for the prevention of CPV-associated disease.

## Introduction

Canine parvovirus (CPV), which belongs to the genus *parvovirus*, is a 20–25 nm-diameter particle consisting of three structural proteins, VP1, VP2 and VP3, with VP2 being far more abundant than the other two proteins. VP2 also represents the major determinant of host range and virus-host interactions. The virion contains a single-strand DNA genome of approximately 5 kb and is encapsulated by a non-enveloped icosahedral particle [1]–[3].

CPV is a highly contagious infectious virus of dogs and can spread to all tissues through the blood. The major consequences of CPV infection are fatal myocarditis in 2–3 week old pups and hemorrhagic enteritis [4]. CPV is of significant economical importance as it can cause large losses in breeding farms. Antibodies play an important role in the protection of dogs against CPV. It is known that a hemagglutination inhibition (HI) of >1∶80 can protect dogs from CPV infection [5]. Vaccines against CPV have been developed from both live attenuated and inactivated CPV strains [6]–[8]. However, the process of growing the virus in established cell lines, its purification, and inactivation or attenuation can be laborious and expensive. In addition, incomplete inactivation of the virus or reversal of an attenuated vaccine strain to a virulent state can cause disease in vaccinated animals [9]. These problems warrant the development of alternative vaccines.

Virus-like particles (VLPs) have been generated and used as vaccine candidates for a variety of viruses due to their ability to induce humoral and cellular immune responses [10], [11]. Previous studies have shown that parvovirus-like particles can be successfully constructed and that they demonstrate excellent immunogenicity [12], [13]. In this study, we used the baculovirus expression system to produce CPV VP2 protein in silkworm pupae to investigate it as a potential vaccine antigen. In addition, the expression of the target protein, the correct assembly of parvovirus-like particles *in vitro*, and the immunogenicity of the particles in mice and dogs were investigated.

## Materials and Methods

### Materials

Silkworms (JY1) were supplied by the Sericultural Research Institute, Chinese Academy of Agricultural Science and were reared on mulberry leaves under standard conditions at 27°C [14]. The vector pFastBac1, *Esherichia coli* DH10Bac/BmNPV (Bombyx mori nuclear polyhydrosis virus) and BmN cells were provided by Jiangshu University of Science and Technology, China. BmN cells, originating from insect ovaries, were cultured in TC-100 insect cell culture medium (Invitrogen, Carsbad, USA) with the addition of 10% fetal bovine serum (FBS, Invitrogen) at 27°C.

### Construction and isolation of recombinant baculovirus

The VP2 gene of CPV was amplified and cloned into the *Bam*HI and *Hind*III sites of the pFastBac1 to generate a recombinant transfer plasmid pFastBac1-VP2. Then pFastBac1-VP2 was transformed into *E. coli* DH10Bac/BmNPV competent cells. The *E. coli* DH10Bac cells containing the recombinant bacmid were propagated in Luria Bertani medium containing 50 µg/ml kanamycin, 7 µg/ml gentamicin, 10 µg/ml tetracycline, 100 µg/ml X-gal and 40 µg/ml IPTG (Isopropyl β-D-1-thiogalactopyranoside, Sigma). The recombinant bacmids were extracted and characterized by PCR. BmN cells at 1×10^6^ cells/well were transfected with 2∼3 µg recombinant bacmid DNA using lipofectamine 2000 transfection reagent (Promega, Madison, USA) according to the manufacturer's instructions. The supernatant containing recombinant baculovirus was harvested from the BmN cells 96 h post-transfection. Titers of the baculovirus were determined by a plaque assay.

### Expression of VP2 protein confirmed by Western blotting and indirect immunofluorescence

About 4×10^5^ pfu of the recombinant baculovirus were injected into silkworm pupae with a needle (26 gauge) and syringe. The infected pupae were collected on day 5, ground in bicarbonate buffer (15 mM Na_2_CO_3_, 35 mM NaHCO_3_, pH 7.2) and centrifuged at 9000×*g* for 15 min at 4°C. The supernatants were collected and stored at −20°C for protein expression analysis (Western blot) and VLP purification by ultracentrifugation.

#### Western blot assay

VP2 protein expression was confirmed by Western blot. The supernatants of samples, which were purified as described above, were mixed with loading buffer and separated by 10% sodium dodecyl sulfate polyacrylamide gel electrophoresis. The separated proteins were transferred onto polyvinylidene fluoride membranes (Invitrogen) and blocked with 1% BSA (Invitrogen) for 2 h. After washing in PBST (PBS plus 0.05% Tween-20) the membranes were incubated with a 1∶300 diluted monoclonal mouse anti-CPV antibody (produced in-house) for 2 h at 3°C. The membranes were washed again, and following a 2 h incubation with a horseradish peroxidase-labeled goat IgG anti-mouse antibody (Boshide, Wuhan, China) the membranes were visualized using DAB (3,3′-diaminobenzidine) solution (Boshide, Wuhan, China).

#### Indirect immunofluorescence

BmN cells (1×10^6^ cells/ml) were grown at 27°C in TC-100 medium supplemented with 10% FBS and inoculated with the recombinant baculovirus Bac-VP2 (1×10^5^ pfu). At 2 days post infection, the 6 well culture plates were fixed with cold acetone at −20°C for 2 h, washed with PBST, and then incubated with an in-house mouse anti-CPV monoclonal antibody (diluted 1∶150 in PBST) and Evans Blue (diluted 1∶500 in PBST) at 37°C for 2 h. After washing, the plates were stained with FITC-labeled goat anti-mouse IgG (1∶150 diluted in PBST) at 37°C for 2 h. Following three more washes, the plates were examined under a fluorescent microscope.

### Vaccine preparation

Purification of CPV-VLPs. The supernatants of samples described above (see 2.3) were precipitated by a 30% saturated solution of ammonium sulfate (pH 7.0). The protein pellet was recovered by centrifugation at 10,000× *g* for 10 min, resuspended in PBS (pH 7.2), and then subjected to sucrose density gradient centrifugation at 35,000 rpm (210053× *g*) in an SW41 Ti rotor (Beckman) for 2.5 h at 4°C. The gradients were fractionated, and fractions that contained VLPs (confirmed by Western blot) were pooled and centrifuged for 2 h at 41,000 rpm (288,244× *g*) in an SW41 Ti rotor to remove the sucrose. The precipitate was resuspended in PBS, and VLPs were visualized by negative-stain electron microscopy.

#### Preparation of oral vaccine

Silkworm pupae were harvested at 5 days post-infection and homogenized by grinding in 4 ml of phosphate-buffered saline (pH 7.2, 15 mM) on ice for 5 min per gram of silkworm pupae. Homogenates were immediately titered by the hemagglutination (HA) test to calculate the hemagglutination unit (HAU) they contained. The raw homogenates were administrated to dogs via the oral route.

### Murine lymphocytes isolation for flow cytometry analysis

BALB/c mice (n = 9) were inoculated intramuscularly with 50 µg of purified VLPs. A control group of nine mice were immunized with 50 µg of total protein isolated from silkworm pupae that were not infected with the recombinant baculovirus. Mice (n = 3) were sacrificed and their spleens and lymph nodes were collected at days 3, 6 and 9. Red blood cells were lysed using an ammonium chloride lysis solution (150 mM NH_4_Cl). Single cell suspensions were then prepared to concentrations of 10^6^ cells/ml in PBS containing 2% FBS and 0.1% NaN_3_. The cells were stained with antibodies (BD Bioscience) against CD3, CD4 and CD8 for 30 min at 4°C. Single or double staining was performed on ice. Data on CD4 and CD8 proliferation were acquired with a LSR-II flow cytometer (BD Bioscience) and analyzed using FlowJo software (Tree Star).

### Animals and experimental design

#### Serum neutralization test (SNT)

Mice (n = 6) were immunized intramuscularly with 70 µg of CPV-VLPs and boosted again with the same dose two weeks later. Three mice were bled at random on the second, fourth, sixth and eighth week post immunization. Neutralizing antibody titers were determined for CPV. The mouse sera were inactivated at 56°C for 30 min, and then diluted serially (2-fold) and mixed with equal volumes of CPV virus containing 100 TCID_50_ [15] in 96-well tissue culture plates. The plates were incubated at 37°C for 1 h and then 100 µl of a feline kidney F81 cell suspension (0.5×10^6^ cells/ml) was added and incubated for 4 or 5 days in order to assess the cytopathic effect (CPE). The highest dilution of sera showing complete inhibition of CPE was taken as the neutralization titer.

#### Hemagglutination inhibition (HI) test

In total six dogs were randomly separated into two groups, one group (n = 3) were orally administrated with 2 ml of raw homogenates containing 4×10^4^ HAU of VLPs. The other group (n = 3) were treated as negative controls and were orally administrated 2 ml of raw homogenates of normal silkworm pupae. Two weeks later, dogs were boosted with the same doses and via the same route. Blood samples were collected from the neck vein pre-immunization and weekly up to 10 weeks post immunization.

Serum samples were inactivated at 56°C for 30 min, then serially diluted two-fold (25 µl of serum) in phosphate-buffered saline (pH = 7.2, 15 mM) in V-96-well plates. Subsequently 25 µl of 8 HAU of CPV were added. The samples were then incubated at 37°C for 1 h after which 50 µl of 1% (v/v) pig erythrocytes were added and the plates were incubated at 4°C for 1 h. Titers were expressed as the reciprocal of the highest serum dilution that completely inhibited hemagglutination. Geometric means were also calculated.

### Hemagglutination (HA) test

HA tests were carried out at room temperature using 1% (v/v) of pig erythrocytes containing 0.1% (w/v) of BSA. Serially two-fold dilutions using 25 µl of homogenates were made in PBS (pH 7.2, 15 mM) in V-96-well plates. Then 25 µl of PBS was added to each well for 1 h at 37°C. Subsequently 50 µl of 1% (v/v) pig erythrocytes were added and the plates were incubated at 4°C for 1 h. The HA titer was determined as the highest dilution of homogenates showing hemagglutination.

### Animal experimentation ethics

In total six dogs age between 12 and 15 weeks were used in this study. They were supplied by commercial dog farm named Kylin and housed in animal facilities with free access to water and food. No CPV vaccine had been administrated before.

All housing conditions and experimental procedures (mice and dogs) were approved by Animal Care and Use Committee of the Chinese People's Liberation Army (No:SYXK2009-045) and were conducted in accordance with the ethical guidelines of the International Association for the Study of Pain.

## Results

### Expression of VP2 in insect cells and silkworm pupae

**Figure 1:**
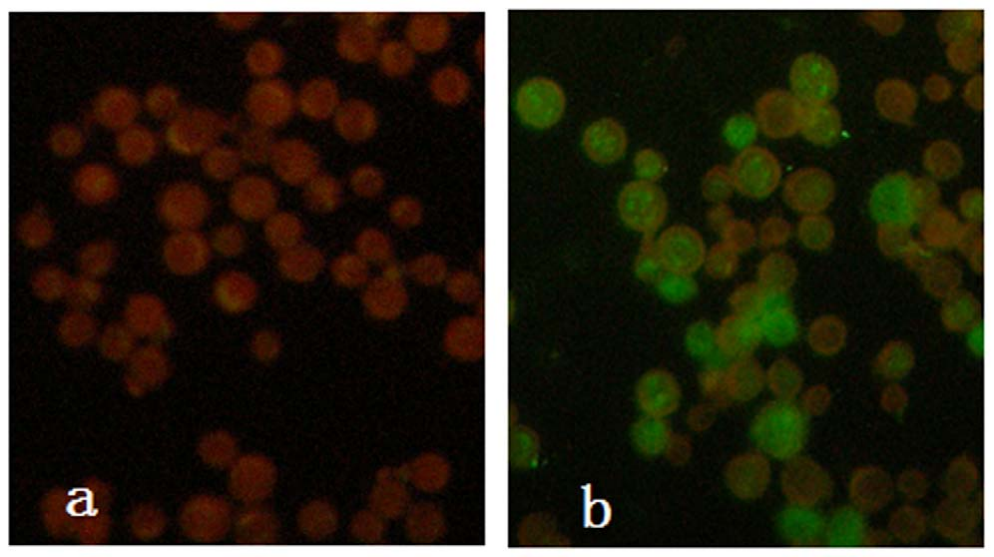
Indirect immune fluorescent antibody assay of VP2 protein expressed in BmN cells infected with recombinant baculovirus. A, Mock-infected BmN cells, B, Recombinant baculovirus infected BmN cells.

**Figure 2:**
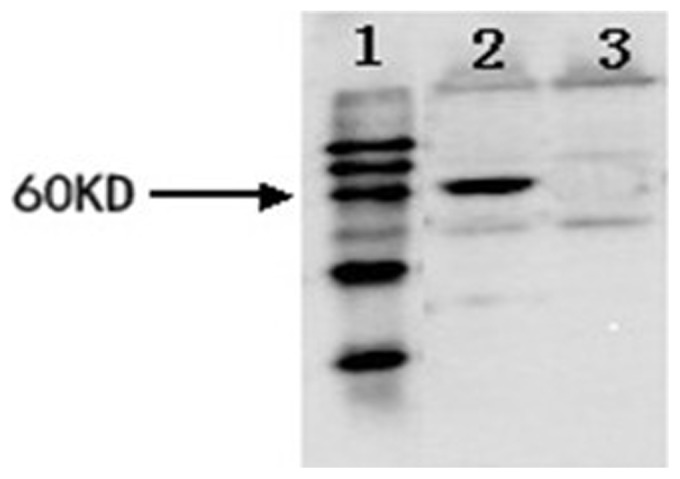
Western blot analysis of VP2 protein expression in the silkworm pupae. Lane 1, prestained protein marker; lane 2, silkworm pupae infected by recombinant baculovirus; lane 3, silkworm pupae infected by wild type baculovirus; numbers on the left indicate the position of protein size markers.

The expression of VP2 in BmN cells was confirmed by immunofluorescence using a mouse anti-CPV monoclonal antibody and FITC-labeled goat anti-mouse IgG at two days post-infection with the recombinant baculovirus (Fig. 1). The schematic representation of VP2 constructs expressed in the pupae using the baculovirus expression system is shown in Figure 2. Pupae infected with recombinant baculovirus were collected at 5 days and subjected to Western blot analysis using monoclonal antibodies directed against CPV. The monoclonal antibody recognized a protein with an apparent molecular weight of 67 kDa corresponding to VP2. These results show that proteins of the correct weight were expressed in the insect cells and silkworm pupae.

### The formation and hemagglutination of parvovirus-like particles

**Figure 3:**
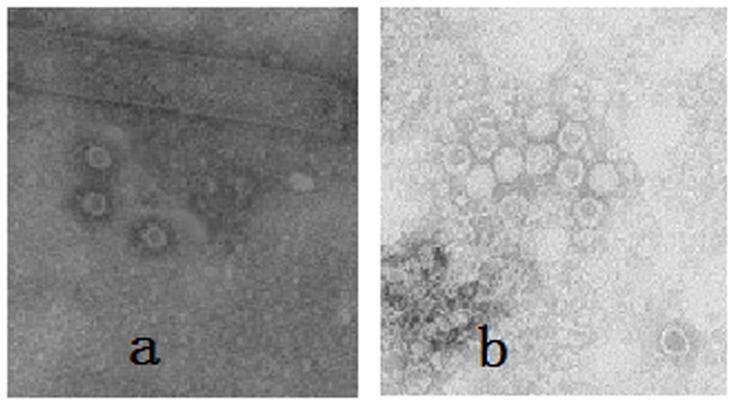
Electron microscopy of CPV-VLP. Sample were negatively stained with 2% uranyl acete and observed by electron microscopy. A, Analysis of the particles formed in recombinant baculovirus infected silkworm pupae. B, Particles collected from sucrose gradients (purified VLP). Magnification 40,000×.

**Figure 4:**
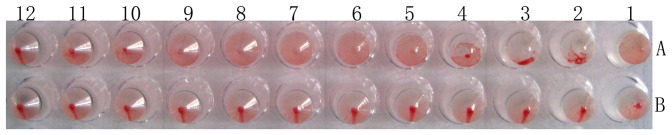
Silkworm pupae homogenates hemagglutinate pig erythrocytes. A, Recombinant baculovirus infected silkworm pupae homogenates. B, Normal silkworm pupae homogenates.

To investigate whether parvovirus-like particles could be formed correctly, the infected samples of BmN cells and pupae were analyzed by electron microscopy. The micrographs clearly showed that the VP2 protein expressed in the cells and pupae self-assembled into parvovirus-like particles with diameters of about 25 nm (Fig. 3a). VLPs were purified by sucrose gradient centrifugation and also examined by electron microscopy (Fig. 3b). Subsequently, hemagglutination of VLPs in silkworm pupae was investigated. Figure 4 shows that VLPs expressed in silkworm pupae could hemagglutinate pig erythrocytes as well as the CPV could and that the titer reached as high as 1∶2^9^. In contrast the normal silkworm pupae homogenates did not show hemagglutination.

### Immunization of mice with CPV-VLPs induced proliferation of CD4^+^ and CD8^+^ T cells

**Figure 5:**
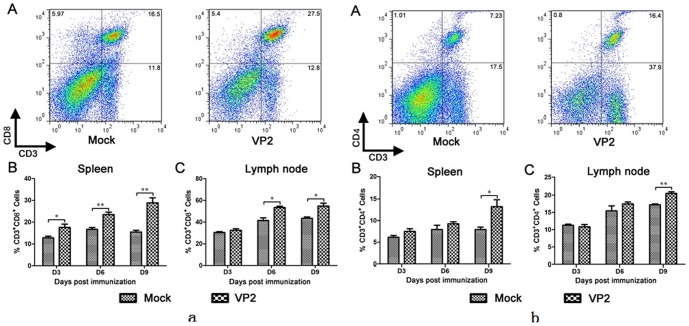
Detection of specific CD4+ and CD8+ Tcell responses. The Lymphocytes from lymph nodes and spleen were isolated at 3, 6 and 9 days post-immunization. Lymphocytes were recovered and stained with cell surface markers CD3, CD4 and CD8. The stained cells were analyzed by flow cytometry (a,b). A, Representative flow cytometric plots of lymphocytes in lymph nodes and spleen at day 9 post immunization. B, All date are from n = 3 mice in each group and presented as mean values±standard errors(SE). Asterisks indicate significant differences between the experimental groups: *, p<0.05.

As shown in Figure 5, in mice immunized with CPV-VLPs, CD8^+^ T cells proliferated in spleens and lymph nodes at 3 days post immunization compared to the mock infected group (Fig. 5a). Furthermore, CD4^+^ T cells clearly proliferated in spleens and lymph nodes at 9 days post immunization compared to the mock infected group (Fig. 5b). These results show that the VLPs produced in the silkworm pupae were highly effective at stimulating CD8^+^ and CD4^+^ T cell proliferation.

### Antibody titer estimation against CPV in mice by SNT

**Figure 6:**
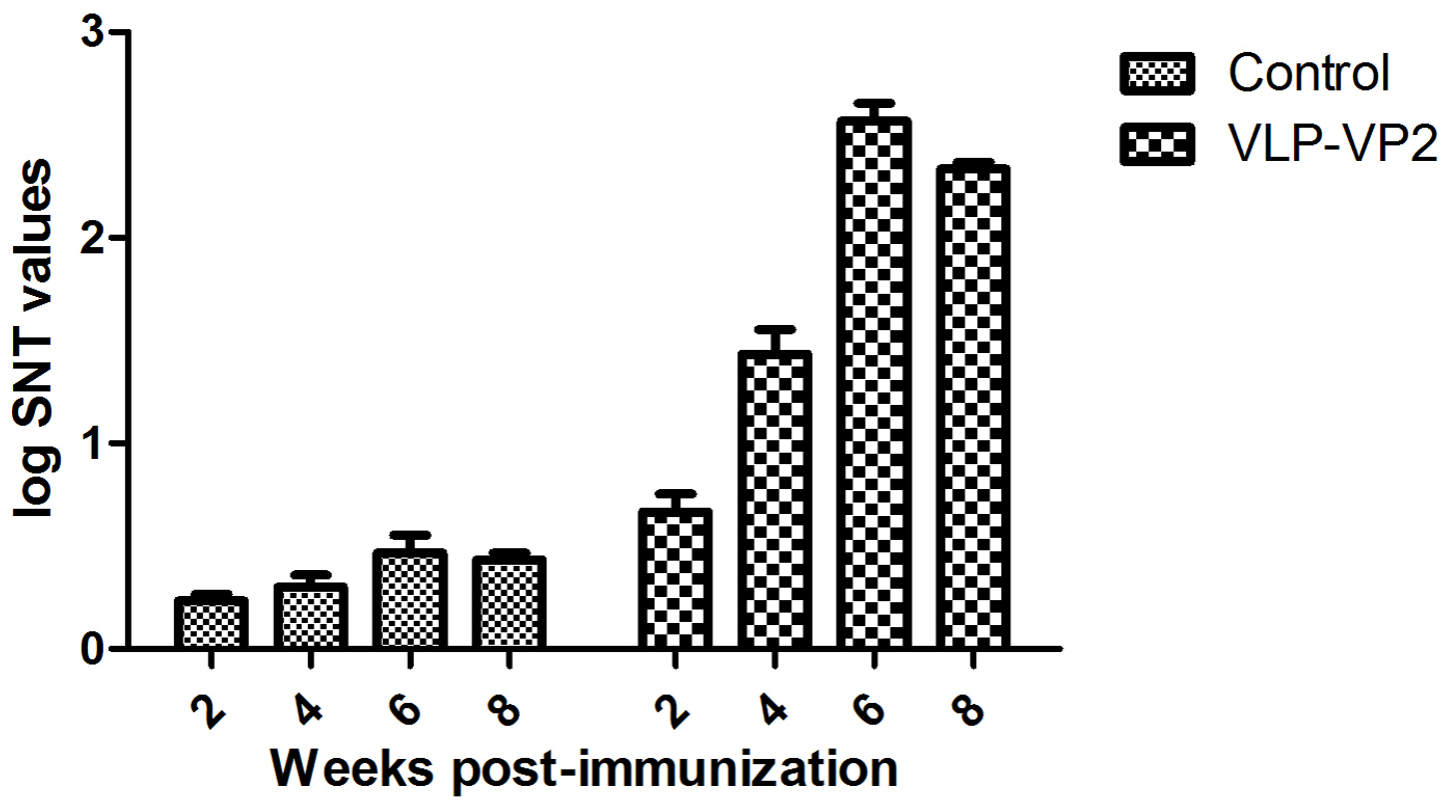
Immunogenicity of VLP-VP2 in mice (determination of antibody titers of VLP-VP2 in mice).

Serum samples collected from mice were subjected to SNT to determine the antibody titer against CPV (Fig. 6). The titer of neutralization antibodies against CPV gradually increased from the second week after vaccination. Following a booster vaccination the antibody titers showed a clear increase which were maintained up to the eighth week post-immunization.

### Antibody titer estimation against CPV in dogs

**Figure 7:**
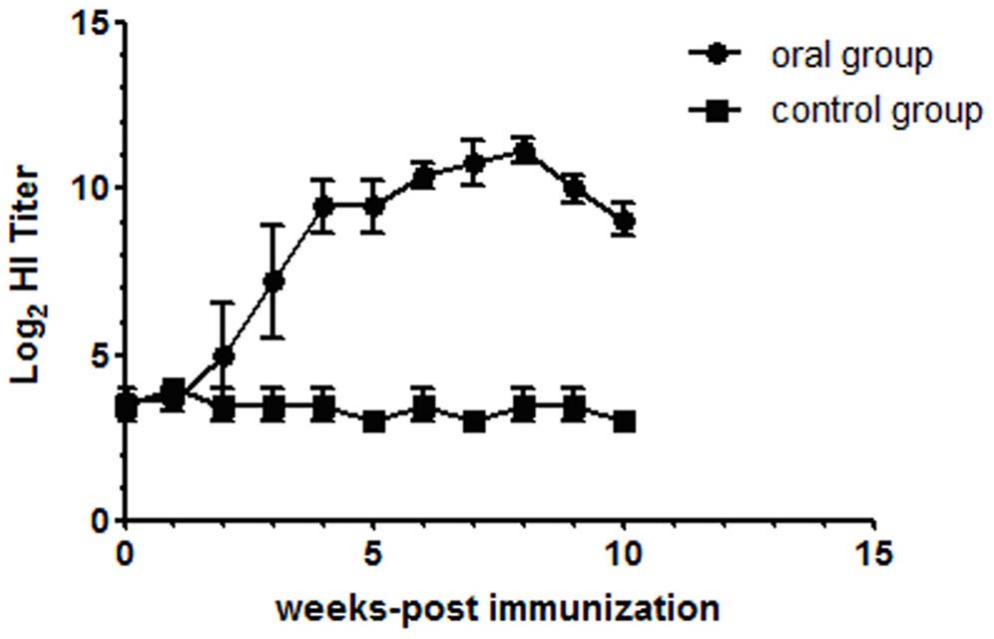
Immunogenicity of VLPs in dogs. Serum was analysed by HI.

Serum samples collected from dogs were subjected to the HI test to determine the antibody titer against CPV (Fig. 7). The HI titers gradually increased from the second week after vaccination. The mean pre-vaccination titer did not differ between the vaccine and control groups. All dogs that were orally immunized with VLPs developed serum IgG anti-CPV antibodies. The rise in serum HI titer was maintained for up to eight weeks.

## Discussion

VLPs possess the same conformation as wild-type viral capsid proteins and are appealing as vaccine candidates because they function as effective antigens without possessing the viral genome or other potentially toxic viral gene products. These particles are highly immunogenic and are able to stimulate both cellular and humoral responses [16]–[19]. Presently, VLPs have been produced for more than 30 different viruses that infect humans and animals [20]. In America, the VLP-based vaccines against human papilloma virus and hepatitis B virus have been approved for clinical use [21], [22]. In addition, trials of Norwalk VLPs are giving promising results [23]. Studies investigating VLPs generated from bursal disease virus, bluetongue virus and influenza virus have all demonstrated the effectiveness of VLPs as vaccines for veterinary use. In this study, we confirmed that the CPV-VLPs elicited both a cellular and humoral immune response and envisage that they could therefore be used as a novel oral vaccine against CPV.

In addition, we successfully demonstrated the expression of VP2 capsid protein of CPV in silkworm pupae using the Bac-to-Bac/BmNPV system. Compared with AcMNPV (Autographa california multiple nuclear polyhedrosis virus), this system can express homologous and heterologous proteins in the silkworm pupae at high levels. The products of VP2 self-assemble into parvovirus-like particles in both insect cells and silkworm pupae. The particles were no distinguishable from wild-type virus in size or morphology, but do completely lack the DNA or RNA genome of the virus. The VLPs also showed hemagglutinating activity when incubated with piglet erythrocytes which is seen for CPV. Taken together, these data indicate that the VLPs produced in the silkworm pupae were similar to the wild-type CPV virion and retained their receptor binding characteristics.

The immunogenicity of VLPs has been well documented by previous studies. For example Ebola virus VLPs were shown to activate mouse bone marrow derived dendritic cells (DCs) as measured by the increased surface expression of CD40, CD80, CD86 and MHC class I and II, as well as the increased secretion of IL-6, IL-10, MIP-1α and TNF-α [24]. Moreover, Balb/c mice immunized with HIV VLPs elicited strong immune responses and were shown to mature monocyte-derived DCs associated with significant up-regulation of CD40, CD80, CD83, CD86 and HLA-DR and increased release of Th1 and Th2 cytokines [25]. In this study, the immunogenicity of VLPs was evaluated in mice and both CD4^+^ and CD8^+^ T cell responses were quantitatively analyzed by flow cytometry. Our results showed that VLPs induced higher CD4^+^ and CD8^+^ T cell responses in mice. The immunogenic ability of VLPs is based on their morphological properties (e.g. size between 20–100 nm and shape), allowing them to be taken up by antigen presenting cells for processing, presentation and promotion of DCs maturation and migration, which are essential for the stimulation of the host's innate immune responses [26]–[31].

VLPs are also able to stimulate B-cell-mediated immune responses. A previous study reported that a single immunization with 0.7 µg of porcine parvovirus (PPV) VLPs yielded complete protection in target animals against infectious PPV isolates [32]. Other studies demonstrated that blue tongue virus (BTV) VLPs resulted in high levels of neutralizing antibodies against homologous BTV serotype in sheep [33], [34]. In this study, mice were immunized intramuscularly with 70 µg of CPV-VLPs without adjuvant. These mice were boosted with the same inoculums after 14 days. All of the mice from the vaccinated groups showed antibody responses to CPV, producing high levels of neutralizing antibodies.

Analogously, after two doses of 4×10^4^ HAU VLPs without adjuvant via the oral route, all dogs developed a system immune response against the VLPs which demonstrated that the VLPs are immunogenic when presented to the gut mucosal immune system. By week four following the first immunization, the serum HI titer was >1∶80 which was sufficient to protect all the immunized dogs from CPV infection. Even without an adjuvant, the VLPs elicited a strong humoral immune response that might be explained by the fact that the VLPs are of a size that can be taken up by DCs and so may possess a self-adjuvanting characteristic. For oral vaccination, gastric acid and enzymatic digestion are major concerns, since they may interfere with vaccine absorption. Our study demonstrated that CPV-VLPs can withstand gastric acid and remain immunogenic.

With regard to the use of VLPs as oral vaccine, this strategy does not require a protein purification process. The silkworm pupae homogenates were safe when used in dogs and the baculovirus does not need to be inactivated. Although the VLPs are immunogenic, the appropriate doses of VLPs and the level of IgA at mucosal secretions (e.g. nasal, oral and fecal) as well as the cytokines should be investigated further. The immune response might be improved if the VLPs possessed a specifically tailored epitope that could target DCs to enhance their ability of antigen processing. In short, to make these VLPs into a feasible and practical oral vaccine, additional research is required.

In summary, VLPs represent a new type of subunit vaccine that are able to stimulate efficient cellular and humoral immune responses against viruses. Our study describes the high immunogenicity of parvovirus-like particles produced in silkworm pupae using a Bac-to-Bac system. These results suggest that, following further studies, CPV-VLPs might be a safe, convenient and effective vaccine for preventing diseases associated with CPV. The use of a baculovirus expression vector system to produce VLPs is attractive. Advantages of this system are low-cost, convenience, and the capacity to produce large and multiple proteins in addition to possessing co-translational and post-translational modifications present in mammalian cell-based systems, including protein glycosylation and phosphorylation and processing [35].
